# Supplementary material for: Unveiling the adoption of metaverse technology in Bangkok metropolitan areas: A UTAUT2 perspective with social media marketing and consumer engagement
Source: PLoS One. 2024 Jun 7;19(6):e0304496. doi: 10.1371/journal.pone.0304496 (PMC11161105; doi:10.1371/journal.pone.0304496)
Supplement: S1 File — (DOCX) [file pone.0304496.s001.docx]

**If you acknowledge and understand the explanation from the researcher. You will receive a questionnaire link.**

**Please mark ✓ in the ❏ in the online questionnaire link.**

❏ Agree to take the questionnaire

❏ Do not consent to take the questionnaire (end of questionnaire)

**Section 1: Screening questions**

1. What kind of activities have you done? Some of the following (You can answer more than 1 question)

( ) Meet/Chat/Share/Review with people on social media such as Facebook, Instagram, Twitter, TikTok

( ) Play online games like fencing or Minecraft on your Nintendo switch

( ) Buy online products to try them in your home through various applications on mobile phones

( ) Study online/meeting/training/chit-chatting/doing activities through the Zoom program

( ) Use smart glasses/VR 3D glasses to play games or explore various virtual worlds

( ) Play games to make money or buy and sell digital assets based on games such as Play-To-Earn NFT/Crypto Games

( ) Doing business, advertising, being a land broker in the virtual world

( ) Travel to various places in the virtual world, such as going to Loi Krathong festival in Metaverse Thailand.

( ) Never performed any of the activities listed above

2. Are you 18 years or older

( ) Yes ( ) No

(End of questionnaire)

**Section 2: Personal Information**

Please put ✓ in ( ) in every answer that most closely matches your opinion.

1. Gender

( ) Male ( ) Female ( ) LGBTQ+

2. Age

( ) 18 – 21 years ( ) 22 – 25 years ( ) 26 – 29 years

( ) 30 – 33 years ( ) 34 – 37 years ( ) 38 – 41 years

( ) 42 – 46 years ( ) 47 – 50 years ( ) 51 years and over

3. Average monthly income (baht)

( ) below 5,001 ( ) 5,001 - 10,000 ( ) 10,001 - 15,000

( ) 15,001 - 20,000 ( ) 20,001 - 25,000 ( ) above 25,000

4. Education level

( ) Below bachelor's degree ( ) Currently studying at the bachelor's degree level

( ) Bachelor's degree ( ) Master's degree

( ) Doctoral degree

5. Marital status

( ) Single ( ) Married ( ) Divorced

6. Zip code of your current address

(specify)...............................................

7. Province of residence

( ) Bangkok ( ) Samut Prakan ( ) Samut Sakhon

( ) Nakhon Pathom ( ) Nonthaburi ( ) Pathum Thani

8. Occupation

( ) Student ( ) Government official

( ) Business owner ( ) State enterprise employee

( ) Private company employee ( ) Freelancer

( ) Farmer ( ) Other, please specify............................

9. Number of family members at current residence

( ) 1 person ( ) 2 – 4 people ( ) 5 or more people

10. Have you ever tried communicating through the metaverse?

( ) Yes, I have ( ) No, I have not

11. What kind of internet technology devices do you use?

( ) Portable notebook

( ) Desktop Computer

( ) Smartphone

( ) Tablet

( ) Other, please specify.................................

12. On average, how many hours per day do you use the internet?

(specify).................... hours/day

**********************

**Section 3: Questions about Metaverse Experience**

**Direction:** Please fill ✓ in the blank space that best describes your opinion in each question (only one answer).

**Question 1**: Performance expectations

| How much do you agree with the following statement about the performance expectations of communication through the metaverse? | Level of opinion | | | | | | | | |
| --- | --- | --- | --- | --- | --- | --- | --- | --- | --- |
| 1. Do you believe that communication through the metaverse is beneficial for your career? | Strongly agree | 7 | 6 | 5 | 4 | 3 | 2 | 1 | Strongly disagree |
| 2. Do you believe that communication through the metaverse accelerates task completion? | Strongly agree | 7 | 6 | 5 | 4 | 3 | 2 | 1 | Strongly disagree |
| 3. Do you believe that communication through the metaverse features beneficial advertisements for users? | Strongly agree | 7 | 6 | 5 | 4 | 3 | 2 | 1 | Strongly disagree |
| 4. Do you believe that communication through the metaverse involves avatars that enhance communication efficiency? | Strongly agree | 7 | 6 | 5 | 4 | 3 | 2 | 1 | Strongly disagree |
| 5. Do you believe that communication through the metaverse helps improve your interactions with other people? | Strongly agree | 7 | 6 | 5 | 4 | 3 | 2 | 1 | Strongly disagree |

**Question 2**: Effort Expectations

| How much do you agree with the following statement about the effort expectations of communication through the metaverse? | Level of opinion | | | | | | | | |
| --- | --- | --- | --- | --- | --- | --- | --- | --- | --- |
| 1. Do you think that communication through the metaverse is easy to use? | Strongly agree | 7 | 6 | 5 | 4 | 3 | 2 | 1 | Strongly disagree |
| 2. Do you think that communication through the metaverse is easy to understand? | Strongly agree | 7 | 6 | 5 | 4 | 3 | 2 | 1 | Strongly disagree |
| 3. Do you think that communication through the metaverse is easy to apply in your profession at work? | Strongly agree | 7 | 6 | 5 | 4 | 3 | 2 | 1 | Strongly disagree |
| 4. Do you think that communication through the metaverse is easy and convenient to use on various devices? | Strongly agree | 7 | 6 | 5 | 4 | 3 | 2 | 1 | Strongly disagree |
| 5. Do you think that communication through the metaverse makes you satisfied? | Strongly agree | 7 | 6 | 5 | 4 | 3 | 2 | 1 | Strongly disagree |

**Question 3**: Social influence

| How much do you agree with the following statement about the social influence of communication through the metaverse? | Level of opinion | | | | | | | | |
| --- | --- | --- | --- | --- | --- | --- | --- | --- | --- |
| 1. Do you think that communication through the metaverse will flourish as more individuals in society accept and utilize it? | Strongly agree | 7 | 6 | 5 | 4 | 3 | 2 | 1 | Strongly disagree |
| 2. Do you anticipate that communication through the metaverse will achieve success as it gains value among people in society? | Strongly agree | 7 | 6 | 5 | 4 | 3 | 2 | 1 | Strongly disagree |
| 3. Do you believe that communication through the metaverse will succeed with the support of executives from different organizations? | Strongly agree | 7 | 6 | 5 | 4 | 3 | 2 | 1 | Strongly disagree |
| 4. Do you believe that your communication in the metaverse will be influenced by the suggestions of those around you? | Strongly agree | 7 | 6 | 5 | 4 | 3 | 2 | 1 | Strongly disagree |
| 5. Do you believe that effective communication within your metaverse will depend on people in society having access to quality internet equipment and technology? | Strongly agree | 7 | 6 | 5 | 4 | 3 | 2 | 1 | Strongly disagree |

**Question 4**: Facilitating conditions

| How much do you agree with the following statement about the facilitating conditions of communication through the metaverse? | Level of opinion | | | | | | | | |
| --- | --- | --- | --- | --- | --- | --- | --- | --- | --- |
| 1. Do you believe you can access your internet account to communicate through the metaverse? | Strongly agree | 7 | 6 | 5 | 4 | 3 | 2 | 1 | Strongly disagree |
| 2. Do you believe you possess knowledge about communicating through the metaverse? | Strongly agree | 7 | 6 | 5 | 4 | 3 | 2 | 1 | Strongly disagree |
| 3. Do you believe you possess a device capable of communicating through the metaverse? | Strongly agree | 7 | 6 | 5 | 4 | 3 | 2 | 1 | Strongly disagree |
| 4. Do you think you can ask for help from other users when communication problems occur through your metaverse? | Strongly agree | 7 | 6 | 5 | 4 | 3 | 2 | 1 | Strongly disagree |
| 5. Do you think that communication via the metaverse has a variety of platforms to choose from? | Strongly agree | 7 | 6 | 5 | 4 | 3 | 2 | 1 | Strongly disagree |

**Question 5**: Emotional motivation

| How much do you agree with the following statement about the emotional motivation of communication through the metaverse? | Level of opinion | | | | | | | | |
| --- | --- | --- | --- | --- | --- | --- | --- | --- | --- |
| 1. Do you think that communication through the metaverse can create an exciting experience? | Strongly agree | 7 | 6 | 5 | 4 | 3 | 2 | 1 | Strongly disagree |
| 2. Do you think that communication through the metaverse can be enjoyable? | Strongly agree | 7 | 6 | 5 | 4 | 3 | 2 | 1 | Strongly disagree |
| 3. Do you think that communication through the metaverse will offer more value than other channels? | Strongly agree | 7 | 6 | 5 | 4 | 3 | 2 | 1 | Strongly disagree |
| 4. Do you think that communication via the metaverse will progressively draw more individuals in society to begin utilizing it? | Strongly agree | 7 | 6 | 5 | 4 | 3 | 2 | 1 | Strongly disagree |
| 5. Do you believe that communication through the metaverse can engender an environment that renders the fictional world three-dimensional? | Strongly agree | 7 | 6 | 5 | 4 | 3 | 2 | 1 | Strongly disagree |

**Question 6**: Price value

| How much do you agree with the following statement about the price value of communication through the metaverse? | Level of opinion | | | | | | | | |
| --- | --- | --- | --- | --- | --- | --- | --- | --- | --- |
| 1. Do you think that product prices in the metaverse should be the same as prices in the real world? | Strongly agree | 7 | 6 | 5 | 4 | 3 | 2 | 1 | Strongly disagree |
| 2. Do you think that product prices in the metaverse should be reliable? | Strongly agree | 7 | 6 | 5 | 4 | 3 | 2 | 1 | Strongly disagree |
| 3. Do you think investing in VR glasses to communicate through the metaverse will be worthwhile in the long run? | Strongly agree | 7 | 6 | 5 | 4 | 3 | 2 | 1 | Strongly disagree |
| 4. Do you find the metaverse top-up system convenient? | Strongly agree | 7 | 6 | 5 | 4 | 3 | 2 | 1 | Strongly disagree |
| 5. Should trading through the metaverse be exempt from taxes, in your opinion? | Strongly agree | 7 | 6 | 5 | 4 | 3 | 2 | 1 | Strongly disagree |

**Question 7**: Habit

| How much do you agree with the following statement about the habit of communication through the metaverse? | Level of opinion | | | | | | | | |
| --- | --- | --- | --- | --- | --- | --- | --- | --- | --- |
| 1. Do you think that communicating through the metaverse will be easy to get used to? | Strongly agree | 7 | 6 | 5 | 4 | 3 | 2 | 1 | Strongly disagree |
| 2. Do you think that communicating through the metaverse might make people more likely to use it for a long time? | Strongly agree | 7 | 6 | 5 | 4 | 3 | 2 | 1 | Strongly disagree |
| 3. Do you think that communication via the metaverse may become necessary in the future? | Strongly agree | 7 | 6 | 5 | 4 | 3 | 2 | 1 | Strongly disagree |
| 4. Do you think that communicating through the metaverse can create an avatar that matches your personality well? | Strongly agree | 7 | 6 | 5 | 4 | 3 | 2 | 1 | Strongly disagree |
| 5. Do you think that communicating through the metaverse allows you to create new identities as you wish in the virtual world? | Strongly agree | 7 | 6 | 5 | 4 | 3 | 2 | 1 | Strongly disagree |

**Question 8**: Social media marketing

| How much do you agree with the following statement about the social media marketing of communication through the metaverse? | Level of opinion | | | | | | | | |
| --- | --- | --- | --- | --- | --- | --- | --- | --- | --- |
| 1. Do you believe discussing the metaverse on social networks will increase awareness among more people? | Strongly agree | 7 | 6 | 5 | 4 | 3 | 2 | 1 | Strongly disagree |
| 2. Do you think creating informative content about the metaverse in online social networks generates interest among technology enthusiasts? | Strongly agree | 7 | 6 | 5 | 4 | 3 | 2 | 1 | Strongly disagree |
| 3. Do you believe celebrity or influencer endorsements contribute to the widespread adoption of the metaverse? | Strongly agree | 7 | 6 | 5 | 4 | 3 | 2 | 1 | Strongly disagree |
| 4. Have people's discussions, exchanges, chats, and shares in online social networking communities made the metaverse a familiar term in this era? | Strongly agree | 7 | 6 | 5 | 4 | 3 | 2 | 1 | Strongly disagree |
| 5. In the realm of various social media marketing efforts, has it encouraged consumers to become more aware of the metaverse? | Strongly agree | 7 | 6 | 5 | 4 | 3 | 2 | 1 | Strongly disagree |

**Question 9**: Consumer participation

| How much do you agree with the following statement about the consumer participation of communication through the metaverse? | Level of opinion | | | | | | | | |
| --- | --- | --- | --- | --- | --- | --- | --- | --- | --- |
| 1. Do you frequently engage with online social network communities? | Strongly agree | 7 | 6 | 5 | 4 | 3 | 2 | 1 | Strongly disagree |
| 2. Do you frequently read posts from online network communities that you regularly follow? | Strongly agree | 7 | 6 | 5 | 4 | 3 | 2 | 1 | Strongly disagree |
| 3. Have you ever provided a review or expressed your opinion in an online networking community? | Strongly agree | 7 | 6 | 5 | 4 | 3 | 2 | 1 | Strongly disagree |
| 4. Do you frequently click to read news about metaverses on websites or online networks? | Strongly agree | 7 | 6 | 5 | 4 | 3 | 2 | 1 | Strongly disagree |
| 5. Would you consider yourself someone who actively keeps up with the digital technology era? | Strongly agree | 7 | 6 | 5 | 4 | 3 | 2 | 1 | Strongly disagree |

**Question 10**: Intention to use

| How much do you agree with the following statement about the intention to use of communication through the metaverse? | Level of opinion | | | | | | | | |
| --- | --- | --- | --- | --- | --- | --- | --- | --- | --- |
| 1. Do you plan to continue engaging in communication via the metaverse in the future? | Strongly agree | 7 | 6 | 5 | 4 | 3 | 2 | 1 | Strongly disagree |
| 2. Do you aim to become recognized as someone who regularly uses metaverse communication in your daily life? | Strongly agree | 7 | 6 | 5 | 4 | 3 | 2 | 1 | Strongly disagree |
| 3. Are you interested in exploring ways to leverage communication through the metaverse for personal benefit? | Strongly agree | 7 | 6 | 5 | 4 | 3 | 2 | 1 | Strongly disagree |
| 4. Do you plan to introduce the use of metaverse communication to people in your social circle when you have the opportunity? | Strongly agree | 7 | 6 | 5 | 4 | 3 | 2 | 1 | Strongly disagree |
| 5. Are you willing to participate in online social network activities to enhance communication through the metaverse? | Strongly agree | 7 | 6 | 5 | 4 | 3 | 2 | 1 | Strongly disagree |

**********************

**Section 4: Additional suggestions**

................................................................................................................................................................................................................................................................................................................................................................................................................................................................................................................................................................................................................................................

............................................................................................................................................................

............................................................................................................................................................

*****End of questionnaire*****

Thank you for your time

**********************
