# Supplementary material for: Unveiling the adoption of metaverse technology in Bangkok metropolitan areas: A UTAUT2 perspective with social media marketing and consumer engagement
Source: PLoS One. 2024 Jun 7;19(6):e0304496. doi: 10.1371/journal.pone.0304496 (PMC11161105; doi:10.1371/journal.pone.0304496)
Supplement: S1 Table — (DOCX) [file pone.0304496.s002.docx]

**S1 Table**. Demographic characteristics of the respondents.

|  | **All** | | **Metaverse use through**  **a notebook computer** | | **Metaverse use through**  **a smartphone** | |
| --- | --- | --- | --- | --- | --- | --- |
|  | **Frequency**  **(n=403)** | **Percentage**  **(%)** | **Frequency**  **(n=194)** | **Percentage**  **(%)** | **Frequency**  **(n=209)** | **Percentage**  **(%)** |
| Gender and sexual orientation |  |  |  |  |  |  |
| Male | 230 | 57.1 | 111 | 57.2 | 119 | 56.9 |
| Female | 164 | 40.7 | 80 | 41.2 | 84 | 40.2 |
| LGBTQ+ | 9 | 2.2 | 3 | 1.5 | 6 | 2.9 |
| Age group |  |  |  |  |  |  |
| 18 - 21 | 80 | 19.9 | 26 | 13.4 | 54 | 19.0 |
| 22 - 25 | 206 | 51.1 | 124 | 63.9 | 82 | 51.1 |
| 26 - 30 | 62 | 15.4 | 29 | 14.9 | 33 | 15.4 |
| 31 – 35 | 22 | 5.5 | 10 | 5.2 | 12 | 5.5 |
| 36 – 40 | 8 | 2.0 | 1 | 0.5 | 7 | 2.0 |
| 41 – 45 | 5 | 1.2 | 1 | 0.5 | 4 | 1.2 |
| 46 – 50 | 5 | 1.2 | 1 | 0.5 | 4 | 1.2 |
| 51 and over | 15 | 3.7 | 1 | 1.0 | 13 | 3.7 |
| Education level |  |  |  |  |  |  |
| Below bachelor’s degree | 24 | 6.0 | 9 | 4.6 | 15 | 7.2 |
| Studying bachelor’s degree | 229 | 56.8 | 130 | 67.0 | 99 | 47.4 |
| Bachelor’s degree | 134 | 33.3 | 51 | 26.3 | 83 | 39.7 |
| Master’s degree | 14 | 3.5 | 3 | 1.5 | 11 | 5.3 |
| Doctoral degree | 2 | 0.5 | 1 | 0.5 | 1 | 0.5 |
| Occupation |  |  |  |  |  |  |
| Student | 254 | 63.0 | 140 | 72.2 | 114 | 54.5 |
| Government official | 12 | 3.0 | 5 | 2.6 | 7 | 3.3 |
| Business owner | 55 | 13.6 | 19 | 9.8 | 36 | 17.2 |
| State enterprise employee | 14 | 3.5 | 12 | 6.2 | 2 | 1.0 |
| Private company employee | 45 | 11.2 | 10 | 5.2 | 35 | 16.7 |
| Freelancer | 9 | 2.2 | 7 | 3.6 | 2 | 1.0 |
| Farmer | 10 | 2.5 | - | - | 10 | 4.8 |
| Others | 4 | 1.0 | 1 | 0.5 | 4 | 1.4 |
| Monthly income (baht) |  |  |  |  |  |  |
| Below 5,000 | 150 | 37.2 | 127 | 65.5 | 23 | 11.0 |
| 5,001 – 10,000 | 33 | 8.2 | 9 | 4.6 | 24 | 11.5 |
| 10,001 – 15,000 | 46 | 11.4 | 2 | 1.0 | 44 | 21.1 |
| 15,001 – 20,000 | 62 | 15.4 | 12 | 6.2 | 50 | 23.9 |
| 20,001 – 25,000 | 33 | 8.2 | 15 | 7.7 | 18 | 8.6 |
| Above 25,001 | 79 | 19.6 | 29 | 14.9 | 50 | 23.9 |
| Family household size | |  |  |  |  |  |
| 1 | 63 | 15.6 | 50 | 25.8 | 13 | 6.2 |
| 2 – 4 | 280 | 69.5 | 124 | 63.9 | 156 | 74.6 |
| Above 5 | 60 | 14.9 | 20 | 10.3 | 40 | 19.1 |
| Province of residency |  |  |  |  |  |  |
| Bangkok | 114 | 28.3 | 51 | 26.3 | 63 | 30.1 |
| Nakhon Pathom | 101 | 25.1 | 52 | 26.8 | 49 | 23.4 |
| Pathum Thani | 49 | 12.2 | 31 | 16.0 | 18 | 8.6 |
| Samut Sakhon | 28 | 6.9 | 4 | 2.1 | 24 | 11.5 |
| Samut Prakan | 66 | 16.4 | 49 | 25.3 | 17 | 8.1 |
| Nonthaburi | 45 | 11.2 | 7 | 3.6 | 38 | 18.2 |
