# Supplementary material for: Unveiling the adoption of metaverse technology in Bangkok metropolitan areas: A UTAUT2 perspective with social media marketing and consumer engagement
Source: PLoS One. 2024 Jun 7;19(6):e0304496. doi: 10.1371/journal.pone.0304496 (PMC11161105; doi:10.1371/journal.pone.0304496)
Supplement: S2 Table — (DOCX) [file pone.0304496.s003.docx]

**S2 Table**. Higher order construct (UTAUT2)

| **Higher-order construct** | **Convergent**  **validity** | **Formative**  **indicators (LOCs)** | **Bootstrap**  **Mean (**β**)** | **Bootstrap**  **weight** | **VIF** | **t-value** | **[LLCI, ULCI]** |
| --- | --- | --- | --- | --- | --- | --- | --- |
| UTAUT2 | Boostrap Mean = 0.809 | PE | 0.756 | 0.174 | 2.145 | 14.929 | [0.152; 0.196] |
|  | R^2^ = 0.991 | EE | 0.827 | 0.113 | 2.893 | 8.229 | [0.085; 0.138] |
|  | t-value = 37.947** | SI | 0.854 | 0.264 | 2.617 | 21.364 | [0.240; 0.289] |
|  | 95% BCCI = 0.764 to 0.846  Cronbach’s alpha = 0.872 | FC | 0.834 | 0.228 | 2.701 | 18.040 | [0.204; 0.252] |
|  | rho_C_ = 0.908 | HM | 0.882 | 0.174 | 3.260 | 11.805 | [0.144; 0.201] |
|  | AVE = 0.604 | HB | 0.842 | 0.226 | 2.562 | 16.835 | [0.201; 0.252] |
|  | rho_A_ = 1.000 | PV | 0.218 | 0.074 | 1.365 | 8.924 | [0.057; 0.090] |

**Abbreviations**: LLCI, lower-level confidence interval; ULCI, upper-level confidence interval; VIF, variance inflation factor
