# Supplementary material for: Unveiling the adoption of metaverse technology in Bangkok metropolitan areas: A UTAUT2 perspective with social media marketing and consumer engagement
Source: PLoS One. 2024 Jun 7;19(6):e0304496. doi: 10.1371/journal.pone.0304496 (PMC11161105; doi:10.1371/journal.pone.0304496)
Supplement: S3 Table — (DOCX) [file pone.0304496.s004.docx]

**S3 Table**. ANN- RMSE results.

| **Training** |  |  |  | **Testing** |  |  |  |
| --- | --- | --- | --- | --- | --- | --- | --- |
| **Inputs: SMM, UTAUT2, CE** | | |  | **Inputs: SMM, UTAUT2, CE** | | |  |
| **Output: IU** | | |  | **Output: IU** | | |  |
| **N** | **SSE** | **RMSE** |  | **N** | **SSE** | **RMSE** | **Total sample** |
| 282 | 41.755 | 0.3848 |  | 121 | 14.6730 | 0.3482 | 403 |
| 293 | 40.219 | 0.3705 |  | 110 | 20.8920 | 0.4358 | 403 |
| 278 | 46.030 | 0.4069 |  | 125 | 16.6200 | 0.3646 | 403 |
| 293 | 39.653 | 0.3679 |  | 110 | 23.1040 | 0.4583 | 403 |
| 278 | 40.601 | 0.3822 |  | 125 | 15.8280 | 0.3558 | 403 |
| 282 | 41.226 | 0.3823 |  | 121 | 23.4500 | 0.4402 | 403 |
| 292 | 44.840 | 0.3919 |  | 111 | 26.8000 | 0.4914 | 403 |
| 275 | 39.289 | 0.3780 |  | 128 | 17.3160 | 0.3678 | 403 |
| 278 | 45.127 | 0.4029 |  | 125 | 14.9500 | 0.3458 | 403 |
| 281 | 41.942 | 0.3863 |  | 122 | 11.1370 | 0.3021 | 403 |
| Mean | 42.068 | 0.3854 |  |  | 18.4770 | 0.3910 |  |
| SD | 2.420 | 0.0125 |  |  | 4.8745 | 0.0608 |  |
