# Supplementary material for: Unveiling the adoption of metaverse technology in Bangkok metropolitan areas: A UTAUT2 perspective with social media marketing and consumer engagement
Source: PLoS One. 2024 Jun 7;19(6):e0304496. doi: 10.1371/journal.pone.0304496 (PMC11161105; doi:10.1371/journal.pone.0304496)
Supplement: S4 Table — (DOCX) [file pone.0304496.s005.docx]

**S4 Table**. Sensitivity analysis.

| **Sensitive Analysis (Important)** | **UTAUT2** | **CE** | **SMM** |
| --- | --- | --- | --- |
| ANN1 | 1.00 | 0.73 | 0.27 |
| ANN2 | 1.00 | 0.72 | 0.18 |
| ANN3 | 1.00 | 0.52 | 0.22 |
| ANN4 | 0.87 | 1.00 | 0.42 |
| ANN5 | 1.00 | 0.82 | 0.25 |
| ANN6 | 0.79 | 1.00 | 0.34 |
| ANN7 | 1.00 | 0.77 | 0.30 |
| ANN8 | 1.00 | 0.60 | 0.22 |
| ANN9 | 1.00 | 0.73 | 0.24 |
| ANN10 | 1.00 | 0.60 | 0.25 |
| Average Important | 0.97 | 0.75 | 0.27 |
| Normalized Important | 100% | 77% | 28% |
| Rank | 1 | 2 | 3 |
